# Perceptions and experiences of young adults and their healthcare team of the D1 Now type 1 diabetes intervention

**DOI:** 10.1371/journal.pone.0316345

**Published:** 2025-02-21

**Authors:** Elizabeth M. McCarthy, Sean F. Dinneen, Molly Byrne, Eimear C. Morrissey, Dympna Casey

**Affiliations:** 1 Health Behaviour Change Research Group, School of Psychology, National University of Ireland, Galway, Ireland; 2 School of Medicine, National University of Ireland, Galway, Ireland; 3 School of Nursing and Midwifery, National University of Ireland, Galway, Ireland; 4 Centre for Diabetes, Endocrinology and Metabolism, Galway University Hospitals, Galway, Ireland; Sefako Makgatho Health Sciences University, SOUTH AFRICA

## Abstract

**Background:**

Young adults (18–25 years) with type 1 diabetes can have high blood glucose levels, increasing their risk of complications. The D1 Now intervention aimed to improve outcomes, using a young adult-centred approach, comprising three components: an interactive messaging system, agenda setting tool, and support worker. A pilot randomised controlled trial was conducted to assess acceptability and feasibility of this novel intervention.

**Aim:**

To explore perceptions and experiences of young adults and healthcare staff participating in the intervention arms of the D1 Now pilot randomised controlled trial.

**Methods:**

A descriptive qualitative approach using semi structured interviews was used to collect data between May 2020, and January 2021 from sixteen young adults and ten healthcare staff Interviews were conducted online using MS Teams. Thematic analysis was used to analyse the data. A patient and public involvement approach was used with the D1 Now Young Adult Panel deployed from the outset to design and contribute to the study. Both written consent in advance and verbal consent was given by all participants for the online interviews that were video and audio recorded according to individual participant preference.

**Results:**

Themes were developed separately for young adult and healthcare staff participants. Two themes were developed from the young adult data, 1) ‘empowerment’ and 2) ‘perceptions and experiences of the intervention’. One theme was developed from the healthcare staff data, ‘perceptions and experiences of delivering the intervention’. All participants highlighted that the agenda setting tool and the support worker empowered the young adults as they could focus the consultation process on what mattered most to them. However, the interactive messaging system was perceived as unsuitable by many mainly because the technology was outdated. Overall, the perceived impact of participating in the study was positive.

**Conclusions:**

Understanding participants perceptions and experiences of taking part in the D1 Now intervention is crucial. The lessons learnt can be used to further refine and develop the intervention with a view to measuring its effectiveness, in a future definitive trial.

## Introduction

Type 1 diabetes is increasing worldwide [1] and young adults with the condition need to monitor their blood glucose and insulin use while also experiencing other major life changes [2, 3]. These life changes include transitioning into work and university, changing social contexts, and competing priorities all of which may lead to young adults feeling challenged and overwhelmed with self-managing their diabetes [4–10].

A further key transition for young adults at this time is the move from attending paediatric diabetic clinics to adult clinics [7, 9, 11, 12], where there is an increased focus on glucose levels and associated complications, which young adults can find stressful [11] leading to poor clinic attendance and loss to follow up [2, 13, 14]. A study of young adult university students in the UK with type 1 diabetes (n = 584) found that 91% never, or rarely, used medical supports on offer within the university, with many losing contact with their prior diabetes team, increasing the risk of complications [8].

Young adulthood is, therefore, considered a high-risk time for young adults with type 1 diabetes [7, 9, 15] and they need to be supported to self-manage their diabetes effectively to improve their health outcomes [16, 17]. However, the first systematic review of interventions designed to identify, describe and measure the effectiveness of interventions aimed at improving clinical, behavioural and psychosocial outcomes for young adults with type 1 diabetes identified an absence of high quality interventions [15]. The review concluded with a need for a theory based intervention informed by key stakeholders to support and improve self-management in young adults with type 1 diabetes [15]. To that end, the D1 Now theory-based intervention, informed by key stakeholders was developed using a systematic, theoretical, user-centred approach to support self-management and improve outcomes in young adults living with type 1 diabetes [18–20]. Central to this was the D1 Now Young Adult Panel consisting of young adults with type 1 diabetes who contributed to all aspects of intervention development [18–20].

Following the development of the D1 Now intervention, a pilot feasibility and acceptability randomised controlled trial (RCT) was undertaken [21, 22] with an embedded qualitative component, the latter is the focus of this current paper.

## Methods

**Fig 1:**
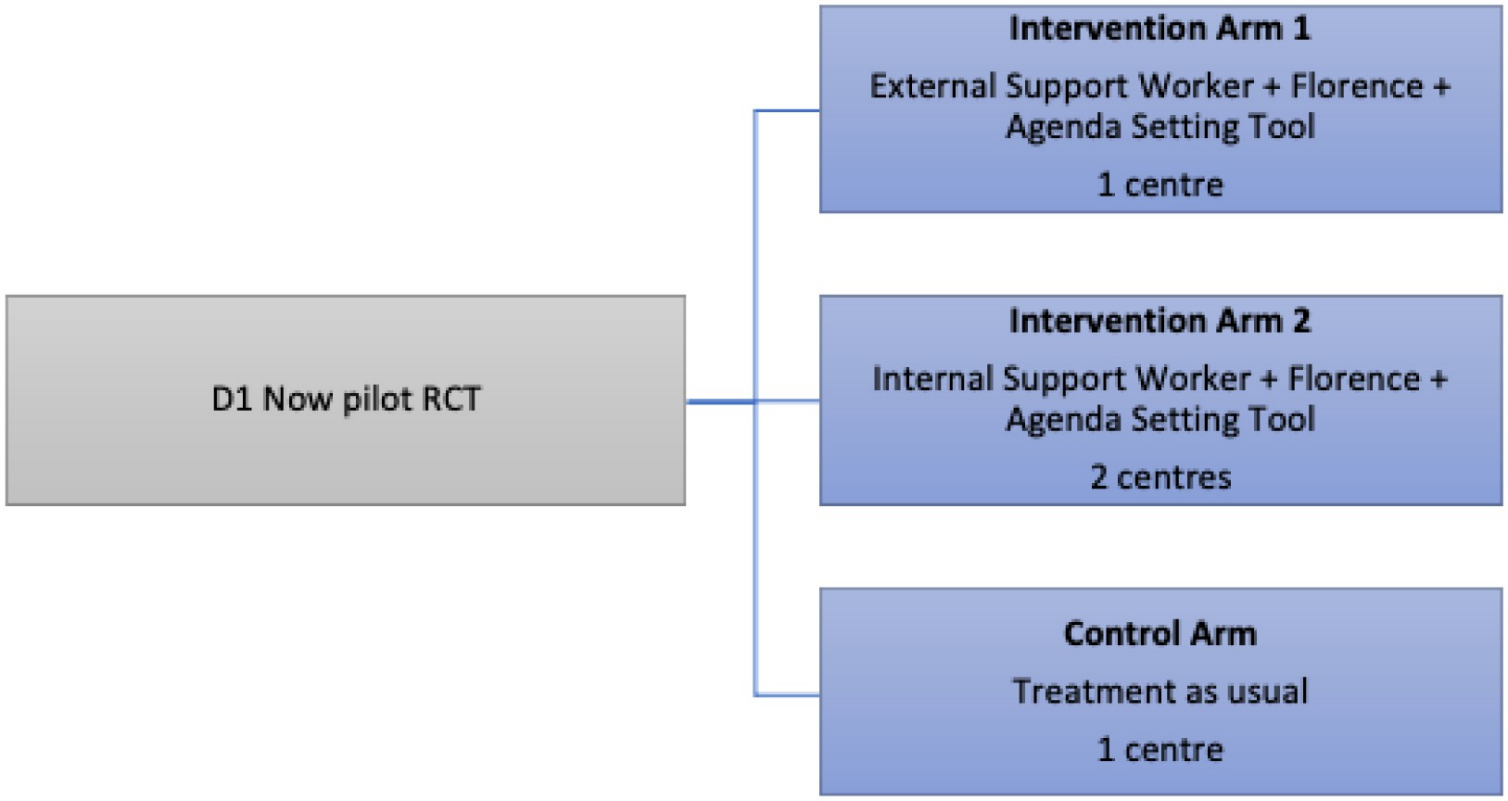
The D1 Now pilot RCT design.

A pilot RCT to refine the D1 Now intervention and determine its acceptability and feasibility for young adults and healthcare staff was undertaken between October 8, 2019, and January 31, 2021 [21, 22] (Fig 1).

### Setting

The D1 Now pilot RCT included four Irish diabetes centres with dedicated young adult clinics: three in the intervention arm and one in the control arm. The three centres randomised to the intervention arm of the pilot trial were invited to participate in this qualitative this study. One centre received the D1 Now intervention with an external support worker (additional staff member hired for the pilot trial) and two centres received the D1 Now intervention with an internal support worker (existing clinical nurse specialists in diabetes who volunteered and upskilled to take on this role).

### Methodology

The D1 Now intervention had three components: 1) a support worker 2) an interactive messaging system (Florence) and 3) an agenda setting tool (AST) (S1 Appendix). The support worker communicated with young adults between appointments by phone, videocall, or email and contributed to multidisciplinary team discussions. The AST contained a question “What do you want to talk about in your clinic appointment today?” and included two screening questions from the Brief Diabetes Distress Scale DDS-2 [23, 24] with the full Type 1 Diabetes Distress Scale (T1-DDS) administered if scores exceeded ‘3’ on the DDS-2 [24, 25]. The software-based text-messaging system called ‘Florence’ also send a prior agreed schedule of reminders and motivational messages to young adults who used it, to assist their diabetes self-management. The intervention is described in detail elsewhere [20, 22].

An embedded qualitative study using a descriptive qualitative approach as described by Sandelowski [26, 27] was undertaken to capture first-hand perceptions and experiences of young adults and healthcare staff of receiving the D1 Now intervention. This approach was chosen to give voice’ to the young adults and the healthcare staff participants views and experiences, enabling the collection of unadorned accounts and facilitating the capture of a descriptive account of findings in everyday language.

### Participants

Fifty-seven young adults took part in the pilot RCT, of which 48 were in the intervention arm. From this group of 48, maximum variation was used to select the sample in relation to age, gender, and study location by intervention site centre to capture a range of experiences. Eighteen young adults agreed to be interviewed. However, two young adults subsequently declined to participate due to work and or study commitments. Further demographic details of the 16 participants are presented in Table 1.

**Table 1:** Demographics of young adult participants (n = 16).

No.	ID Code	Intervention Arm	Age	Age of diagnosis	Insulin administration	Time of interview
1	YA004	External	20	2	Pump	Six months
2	YA012	External	21	7	Pen	Six months
3	YA013	External	22	9	Pen	Twelve months
4	YA019	External	23	14	Pen	Twelve months
5	YA020	External	20	5	Pump	Six months
6	YA021	External	19	10	Pump	Twelve months
7	YA025	External	19	10	Pump	Twelve months
8	YA022	Internal,1	20	7	Pump	Twelve months
9	YA005	Internal,1	23	4	Pump	Twelve months
10	YA018	Internal,1	25	7	Pump	Six months
11	YA016	Internal,1	22	9	Pump	Twelve months
12	YA017	Internal,1	20	12	Pump	Six months
13	YA001	Internal,2	22	6	Pump	Twelve months
14	YA006	Internal,2	20	17	Pen	Six months
15	YA008	Internal,2	26	8	Pump	Six months
16	YA014	Internal,2	20	19	Pen	Six months
	**Total Interviews**			**16**		

Healthcare staff (HCS) from across the three intervention sites, were also invited to interview, (n = 10). To protect at much anonymity as possible due to the small sample and small number of sites, details regarding profession, gender, age, years of experience and the site they were allocated is not presented. Instead, a descriptive overview of participants is provided (Table 2: Description of Healthcare Staff).

**Table 2:** Description of healthcare staff (n = 10).

Average Age	Average Years of experience	Male	Female
46 years	12 years	n = 3 (30%)	n = 7 (70%)

### Data collection

Semi-structured interviews using interview guides (S2 Appendix) were conducted by EMcC with healthcare staff (n = 10) at month 12 and with different young adult participants at month 6 (n = 7) and month 12 (n = 9) to reduce participant burden (see Table 1). All interviews were one-on-one, conducted online using MS Teams and 30–45 minutes in duration. The interview guide for young adults was developed based on the research objectives, expertise of the research team (DC, EM and EMcC) and in conjunction with the D1 Now Young Adult Panel. The interview guide for healthcare staff was also designed by the study team based on the research objectives and expertise of the research team. The arrival of the COVID-19 pandemic in March 2020 impacted the delivery of the D1 Now intervention for about three months, when most diabetes clinics moved to telephone or virtual clinics. Therefore, a question to ascertain the impact of COVID-19 on participation was added to the original interview guide (S2 Appendix). All participants reiterated their consent to participate verbally at the beginning of the interview. The interviews were recorded on MS Teams either using audio only or using both audio and video, depending on participants’ preferences. All data was subsequently transcribed verbatim, personal identifiers removed and uploaded to NVivo12 to facilitate the storage and management of the data.

### Data analysis

Thematic analysis based on the work of Braun and Clarke using the six-phase process [28, 29] was employed which emphasises the active role of the researcher, advocating an organic approach to the coding of data and development of themes. Firstly, familiarisation with the data was achieved by EMcC reading the interview transcripts repeatedly; codes were then generated by highlighting quotes from the transcripts that seemed to capture key concepts across the data (EMcC). A secondary review of the coding was undertaken by DC to ensure consistency. Theme searching was then commenced by EMcC which involved exploring how different codes could be combined to form overarching themes: these themes were then reviewed by DC to ensure that there was enough data to support each theme and that the labels and theme titles applied reflected thematic content. The final stage involved integrating themes in a meaningful way and writing up the narrative [28, 29]. When similar themes were beginning to be repeated during the interviews, two more interviews were undertaken to confirm data adequacy.

The four criteria described by Lincoln and Guba (1985) were used to ensure rigor [30]. Rigor was also ensured by the researcher (EMcC) maintaining a reflective journal during the entire analysis process. In addition, contemporaneous notes were maintained during interview sessions, and when developing codes and final themes. The reflective journalling and note taking process supported self-reflection and helped the researcher to consciously keep track of their thoughts and remain vigilant of potential biases that might influence their interpretation of the data. Ongoing discussion throughout the analysis process with research team members with qualitative expertise and knowledge of diabetes (EMcC, EM, DC) also helped to ensure rigor and to further limit any potential influence and/or bias by the interviewer during interviews and data analysis. Finally, members of the D1 Now Young Adult Panel also reviewed a selection of anonymized transcripts and confirmed that the interview transcripts were reflective of their own experiences.

### Ethics

Ethical approval was obtained from the ethical committees of each of the three hospitals participating in the study: Beaumont Hospital Ethics (Medical Research) Committee REC reference: 19/51; St. Vincent’s University Hospital Local Ethics Committee Ref. No: RS19-031; and Tallaght University Hospital Research Ethics Committee Rec: 2019–09 List 35 (13). Participants all received a participation information leaflet and written informed consent was obtained from all participants.

## Results

Two themes were developed from the young adult data, 1) ‘empowerment’ and 2) ‘perceptions and experiences of the intervention’. Following analysis of the healthcare staff data, one theme was developed, ‘perceptions and experiences of delivering the intervention’.

### Young adults

#### Theme 1: Empowerment

This theme describes how the D1 Now intervention empowered young adults during clinic consultations to participate more in the management of their diabetes. It consists of the sub theme, ‘Feeling supported and in control’.

*Feeling supported and in control*. Some young adults reported initial hesitancy about study participation, expecting it to become burdensome and restrictive as they would be obliged to ‘have to’ do several things. Conversely, they reported positive experiences, with the usual busyness of the clinic balanced by being able to discuss their feelings resulting in them feeling more in control of their clinic appointment.

*…at the start*, *I was a bit sceptical…I didn’t want it to become a burden… It was actually completely the opposite*, *I felt like I had a bit of control then*, *rather than owing someone a favour*

*(YA021)*

Many young adults also reported that completing the first page of the agenda setting tool with the support worker, in advance of their consultation, made them more in control and prepared. The first question *‘what would you like to discuss today’* was identified as key, allowing them to focus on what was important to them rather than the needs of the endocrinologist.

*I personally found it very beneficial… getting this new structure and especially…the first question asking*, *"what would you like to discuss today"*

*(YA006)*

Many young adults reported feeling actively included in finding solutions to their problems as working with the support worker helped them to better understand their diabetes and this allowed them to become more involved and collaborate with their healthcare team rather than being managed as previously.

*A support worker…it’s more about the management… rather than like your diabetic nurse…more concerned with taking your bloods and booking appointments…you can kind of start to understand why there’s patterns forming…it’s just that extra support*

*(YA001)*

They also reported that the good relationship developed with the support worker was due to their consistent presence and familiarity with their medical history. This contrasted with prior clinic experiences where they met different healthcare professionals at each visit with some young adults reporting feeling anxious and upset, specifically during meetings with the consultant endocrinologist.

*… the normal clinic*, *you get a different nurse and a different doctor every time…*. *she [support worker] stayed the same and I really like that… somebody knows your medical history …*.

*(YA005)*

*The consultant [endocrinologist] meetings…they are the ones that upset more or cause someone to be frazzled… I’ve definitely gotten side-tracked in consultant meetings a lot more than I would with my nurse*

*(YA006)*

Several young adults referred to diabetes distress as a ‘taboo’ subject, perceiving the focus of consultations to be only on HbA1c. The agenda setting tool, in contrast, gave them control and permission to discuss their distress and consider the impact of their diabetes on both their physical and mental health, which was new.

*[Diabetes distress]… one of those kind of taboo subjects where we don’t really talk about it…*.

*(YA008)*

*[agenda setting tool] like a catalyst*, *almost gave you a starting point for being able to talk about [diabetes distress]…would be the kind of thing that I would be a bit nervous to bring up on my own*

*(YA017)*

*I just kind of like separated diabetes…physical health and mental health were separate*

*(YA013)*

#### Theme 2: Perceptions and experiences of the intervention

This theme describes young adults’ perceptions and experiences of the three aspects of the intervention and as such is reported under the subthemes 1) support worker, 2) agenda setting tool 3) Florence and 4) the impact of COVID.

*Support worker*. Most young adults reported that they viewed the internal support worker not as a nurse but as a friend, someone who knew them and would work with them and help them to manage their diabetes better.

*Having that point of contact is great…knowing them by name*, *and they know you…you don’t kind of see them as like the clinic nurse anymore… you actually feel like they are trying to help…they look at your sugars and like ’what can we do now to sort you out’*

*(YA008)*

Most similarly reported that the presence of the support worker (internal or external) at each clinic appointment was both beneficial and acceptable. Many reported being anxious about their diabetes and appreciated the support worker providing positive reinforcement and helping them to view their diabetes in a different way.

*…lot of anxiety with dealing with my diabetes*. *[Support worker] was quite helpful… a lot of positive reinforcement…looking at my illness in a more progressive light as opposed to kind of always been down in the dumps about it*

*(YA019)*

*Agenda setting tool*. The majority of young adults also reported that the agenda setting tool was acceptable and easy to use, providing focus and highlighting the need to be more mindful of their diabetes.

*…it [agenda setting tool] really helps make you more conscious of… things that are going on with your diabetes*

*(YA005)*

This tool was perceived by most young adults as having the most positive impact of the D1 Now intervention. They reported that the goal setting exercise component enabled them to identify, discuss, and agree realistic and achievable self-management goals with their consultant, something they had not previously done.

It was interesting to note that when asked if any of the intervention components could standalone, the majority of young adults reported that they felt the agenda setting tool and support worker combination complemented each other and would not work well on their own.

*more beneficial to have the support worker there that can kind of guide you through it [agenda setting tool]…I don’t think they would work [separately]*

*(YA001)*

*Florence*. Most young adults who engaged with Florence reported that they either already had a reminder system in place, found it annoying, or felt overwhelmed with the frequency of text messages hence they switched it off. However, for some young adults knowing they could contact the support worker directly if needed served to decrease their anxiety, even if they did not use it.

*… appreciated it more [Florence/Support worker] because I felt like there was kind of an outlet like if I needed it*

*(YA013)*

While some young adults acknowledged that it might be useful for others, particularly younger people.

*I did [use Florence] for a little while and then they [messages] just kind of annoyed me …I was just getting inundated with messages…I just ignored them… younger [person] or something like that*, *maybe it would be more beneficial*

*(YA019)*

### Impact of COVID on the intervention experience

The onset of COVID in March 2020 resulted in diabetes clinics moving to telephone or virtual clinics. Some young adults described the telephone calls during the COVID lockdown as basic and generic, conducted with unfamiliar staff, with little opportunity to discuss their concerns. However, some acknowledged that being part of the intervention and knowing that they could reach out for help was reassuring.

*…the questions [telephone-call] were just too general … … ‘Are you ok with checking your blood sugars?’…for someone who had diabetes for 12 years*, *I could have done without the call*

*(YA016)*

… *appreciated it more [Florence/Support worker] because I felt like there was kind of an outlet like if I needed it*

*(YA013)*

### Healthcare staff

#### Theme 1: Perceptions and experiences of delivering the intervention

This theme describes healthcare staff perceptions and experiences of delivering the intervention. It consists of three sub themes, 1) rebalancing the power dynamic, 2) resource implications and training and 3) impact of COVID.

*Rebalancing the power dynamic*. Most healthcare staff reported that the agenda setting tool was a welcome change to their current practice serving to rebalance power and refocus the consultation to the young adults wider needs and not just on the ‘numbers’ and that it gave structure to the consultation and helped balance the power dynamic in the consultation process, empowering young adults.

*…I think it gives structure to the clinic…so useful in terms of …generating point that can be followed up over time…it is good I think…so you know it could help clarify things…*

*(HCS2)*

*… it ensures that the consultation is a two-way process addressing the concerns and priorities of both sides*, *rather than a doctor’s perspective of what needs to be discussed*.

*(HCS3)*

The importance of the first question on the agenda setting tool was highlighted as they felt that this ensured that the young adults had time to reflect and create a plan before meeting the consultant.

*I think it was really important to*, *for them to highlight what it is they wanted to specifically talk about*, *and it got*, *they got more I think out of their appointment then*.

*(HCS7)*

*You know*, *it was great to have it a little bit more led by the young person as opposed to us setting the agenda for them*.

*(HSC4)*

*Resource implications and training*. Most healthcare staff reported that the support worker was an acceptable addition to the clinic. However, concerns were expressed by a few staff about the viability of a support worker in its current form due to resource implications and that the lack of resources increased the burden on other staff and was unfair.

*…my chances of [funding support worker] in the next five years are slim…the reasons for really being involved in this project is hopefully to get a bit of data…important to re-apply with a business case*

*(HCS1)*

*…it…meant that some of my other nurses had to carry an extra bit of work…But the core principle …I really like it*, *but the notion of no extra resources*, *now you’re a support worker is unfair on everybody*.

*(HCS3)*

Some of the support workers perceived their role, and the care they provided in the intervention, aligned well with their existing established responsibilities as experienced healthcare staff.

*… It’s not hugely different than what I would have done in my role with the young people in our clinics before… I would have been very much a pivotal*, *central person that they contact*

*(HCS10)*

However, this role did require adjustments for example it increased administrative workload. Furthermore, there was a tendency for staff to direct study participants to them in the first instance regardless of the issue.

*…the hardest part for me has been trying to keep up with the paperwork …if a young person came in who’s one of my recruits*, *then the girls would re-direct it back to me rather than them seeing the person themselves*

*(HCS10)*

Other support workers reported that their main adjustment had to do with finding their way in a new role and different environment.

*… I kind of was in uncharted waters…there was quite a lot of navigating to do at the start of the role…trying to figure out what is it that I’m here to do?… feeling a little bit like an outsider…*

*(HC8)*

While all healthcare staff were extremely positive about the support worker, most felt it needed to be an experienced diabetes educator and communicator, with the skills and training to support the young adult, and familiar with navigating hospital systems. Many also reported that the role needed to be a full-time post with additional resources ring-fenced and secured to ensure its viability into the future.

*… [support worker] has to be an internal person…an additional resource to what’s already available… it’s a full-time post…the patients appreciate it… it needs to be ring fenced*, *secured*, *protected resource*

*(HCS3)*

*…I don’t think we would have been able to facilitate having one of the nurses as the support worker*. *We just wouldn’t have been*, *had the time… So*, *I think you need someone that’s just dedicated to that aspect {Support Person} of it*, *you know*.

*(HCS4)*

Many healthcare staff also raised concerns regarding the resources needed to manage the delivery of Florence as well as its viability as a motivational tool. Florence was described as generic; impersonal with out-of-date technology with many of its reminder features already available on other smartphone apps.

*…I guess I’d have mixed feelings about how useful young people found Florence based on the level of engagement and whether or not actually it may be slightly out of date at the moment as a technology tool…*

*(HCS8)*

Some healthcare staff also acknowledged that the D1 Now intervention highlighted some resource deficiencies in the service in terms of not being able to devote sufficient time to clients and lack of training to deal with issues related to diabetes distress. The latter resulted in staff shying away from the topic as they perceived they were not fully equipped or trained to deal with psychological issues with no care pathway to follow.

*[D1 Now] highlighted to us how our service kind of something’s lacking in our service in that we don’t all have time to sit down and speak to patients in this way and we’re obviously not trained in it…you’re not able to give them a solution… there isn’t a lot of support out there…if I open this can of worms*, *where do I take it?*

*(HC6)*

In contrast, other healthcare staff reported that the initial training provided by the D1 Now study team increased their knowledge and confidence and empowered them in dealing with diabetes distress.

*it’s good to normalise that [diabetes distress] as part of diabetes care and we were given some training at the beginning… so I felt comfortable I suppose with that included as part of the tool and to be discussed in consultations…*

*(HCS5)*

Several healthcare staff reported that they were concerned initially about the added pressure delivering the intervention might have on the smooth running of an already busy clinic. However, they reported that these concerns abated and the experience was very positive.

*…bit of nervousness how would the flow of the clinics go…would it slow down the clinics*, *we’re under huge pressure in the clinics to get the clinics done in a certain time but actually we got through all of that*, *we got over all of that… it’s been very*, *very positive*

*(HCS1)*

### Impact of COVID on the intervention experience

Several healthcare staff viewed the move to telephone or virtual clinics during COVID as less optimal as interactions were short, and sometimes conducted by staff unfamiliar with the study. In this context for example the full diabetes distress scale (T1-DDS) was considered too long to implement.

*We actually didn’t use the D1 Now tool [in virtual consultation] …it wasn’t only the consultant that was doing the virtual clinics…other members of the diabetes team that wouldn’t have been core to the D1 Now young adult clinic group…*

*(HCS8)*

*… [The T1-DDS] 28 questions*! *that was going to take me a minute per question …the way it [COVID-19] has affected the D1 Now is that it hindered the face to face*, *which I think is more useful than any virtual appointment*

*(HCS9)*

In addition, staff absences due to COVID and the amplified demand for patient reassurance during lockdown, also increased staff workload.

*Again*, *COVID has meant that we’ve had staff off*, *you know*, *above the normal quantities at different times and*, *you know*, *so that perhaps magnified the impact of someone trying to take on extra work around a study in this case*,

*(HCS3)*

*Yes*, *there’s been more phone calls*, *there’s more enquiries*, *you know*, *people are phoning us because they’re anxious and they need assurance in some respects and they*, *you know*, *they just need a bit more support…So*, *now you’re making phone calls for clinics sometimes…I’m in a car*, *well now ‘will you ring me back tonight at 8 o’clock’*, *so*, *getting the time…it’s a challenge…*

*(HCS5)*

## Discussion

This qualitative embedded study explored the perceptions and experiences of young adults, and their health care staff who participated in the intervention arm of the D1 Now pilot RCT. All young adults and health care staff reported that two intervention components, the agenda setting tool and support worker combined, were acceptable and viable and a welcome addition to the clinic experience. This was because the tool was perceived as giving structure and meaning to clinic visits and the support worker provided advocacy for the young adults. Specifically, young adults described the agenda setting tool as having the most significant impact, giving them control in the consultations and helping them to talk about and agree realistic and attainable self-management goals that was a new experience. The importance of goal setting to facilitate diabetes self-management and empowerment in young adults is also supported in other studies [31, 32].

Perceptions of the text-messaging service (Florence) however were mostly unfavourable. All health care staff perceived Florence as generic and unsuitable for a young adult population who already have access to multiple types of technology for diabetes management. Both healthcare staff and the young adults alike felt that the device needed to be more customised to young adults’ preferences. These findings concur with a study assessing applications (apps) designed for diabetes self-management that did not work as well as intended. The study concluded they needed to be customisable and embedded with evidence-based information; problem-solving skills; and to support transitioning from paediatric into adult care [32].

The onset of COVID in many countries, as in Ireland, witnessed a shift from face-to-face care delivery to virtual or telehealth care [33, 34]. Some health care professions in this study felt that this change made it difficult to deliver the intervention as they found that the telephone calls tended to be short. This may be due to the fact that this change was suddenly thrust upon health care staff who had little or no training in using this technology to effectively create an atmosphere conducive to open discussions in these new environments [35]. In addition, during COVID many health care staff found themselves redeployed to care for patients in unfamiliar health care contexts and it took a little time for these staff to become familiar with caring for new clients. However, as identified in the literature most nursing and health care staff very quickly adapted [36, 37].

Although several health care staff reported that prior to the commencement of the study, they had concerns about delivering the intervention perceiving that it might increase their workload and delay the flow of young adults through the clinic, this did not materialise. Young adults also reported previously perceiving adult clinics as ‘busy’ and ‘lengthy’, but the D1 Now intervention that facilitated scheduling convenient appointments with their support workers made it an enjoyable experience. These findings concur with previous findings in a study where young adults valued shorter waiting times; flexible appointments; and seeing both the nurse and consultant [38].

Resource issues surrounding the introduction of any health care intervention is an important consideration. Although the experience of the support workers was overall very positive, a concern was raised regarding a perceived increase in their administrative workload with some healthcare staff noting that the role needed to be clarified and time protected. Interestingly, the health economic assessment of the D1 Now intervention relative to usual care (control arm) [22] revealed little difference between the estimated mean cost per patient for internal support workers at €3,755 (SD: 1,762), and external support worker at €3,944 (SD: 1,489).

Diabetes distress describes a person’s perception of the emotional burden of managing their diabetes with higher rates observed in young adults, which may contribute to adverse outcomes [39]. The term ‘diabetes distress taboo’ was used by some young adults in this study relating to both their own discomfort and that of their healthcare provider, in raising the emotional burden of self-management during consultations. Some healthcare staff admitted they were reluctant prior to the D1 Now intervention to ‘open the… can of worms’ due to lack of knowledge and skills: this reluctance was also reported in other studies where upskilling was recommended [40]. Combining a support worker skilled in dealing with diabetes distress, along with the agenda setting tool, was perceived as beneficial by both the young adults and healthcare staff. This novel combination opened conversations, shifted the power dynamic and provided the structure to discuss strategies to support self-management that did not exist prior to this study.

Many young adult participants reported high anxiety levels during previous clinic consultations, prior to the study due to the focus on HbA1C and ‘the numbers’, an issue also highlighted in other studies who reported healthcare staff as overly critical and number-focused [40, 41]. However, in this study the question on the AST ‘what would you like to discuss today?’ was perceived as crucial by young adults, empowering them to shape the consultation to their needs, and not just their HbA1c.

### Strengths and limitations

A possible limitation of this study was the impact of COVID-19 which resulted in young adults clinic appointments being moved online or conducted over the phone. However, all young adults positively evaluated the intervention components apart from Florence, which was identified as annoying and outdated, a finding unrelated to the pandemic.

There was a predominance of female healthcare participants in this study, however, this reflected the workforce gender balance. Additionally, more female young adults than males responded and agreed to participate in the study. That more females than males participate in surveys and interviews is not uncommon and the reasons for same remain unclear [42]. In any future definitive RCT, a gender balanced representative sample will be sought and factors governing reduced participation by either gender will be more fully explored.

A key strength of the study was the involvement and the input of the D1 Now Young Adult Panel who were deployed from the outset, contributing to the development of the interview guides; piloting of interviews; and informing the thematic analysis process. Another strength was the fact that the interviewer (EMcC) was not involved in earlier phases of the RCT, therefore, bias and assumptions were further reduced.

## Conclusion

This embedded qualitative study, a component of the D1 Now pilot RCT, indicates that the presence of a dedicated support worker combined with the agenda setting tool, has the potential to provide more structure and meaning to consultations for both young adults with type 1 diabetes and their healthcare team, thereby positively change clinical practice. However, the impact of these components in particular their effectiveness on young adults’ ability to self-manage requires further testing, in a definitive RCT, prior to any widespread implementation in practice.

## Supporting information

S1 Appendix Agenda setting tool. (DOCX)

S2 Appendix D1 Now interview guides. (DOCX)
