# Supplementary material for: Perceptions and experiences of young adults and their healthcare team of the D1 Now type 1 diabetes intervention
Source: PLoS One. 2025 Feb 21;20(2):e0316345. doi: 10.1371/journal.pone.0316345 (PMC11844834; doi:10.1371/journal.pone.0316345)
Supplement: S1 Appendix — (DOCX) [file pone.0316345.s001.docx]

**Appendix A: Agenda Setting Tool**
