# Supplementary material for: Perceptions and experiences of young adults and their healthcare team of the D1 Now type 1 diabetes intervention
Source: PLoS One. 2025 Feb 21;20(2):e0316345. doi: 10.1371/journal.pone.0316345 (PMC11844834; doi:10.1371/journal.pone.0316345)
Supplement: S2 Appendix — (DOCX) [file pone.0316345.s002.docx]

**Appendix B:** **D1 Now Interview Guide****s**

**Young Adults**

The D1 Now study in which you are taking part is a ‘pilot’ study. This means we are trying to learn as much as we can about which parts of the intervention work well and which don’t work so well before rolling it out on a larger scale. It is very important that we hear what patients’ think about the intervention and about their experiences of D1 Now. Can I check that you are happy to take part in the interview today? Just to remind you, we can stop the interview at any time if you want.

| **Topic** | **Question** | **Prompt** |
| --- | --- | --- |
| **Experience**  What are young adults’ experiences of using the D1 Now intervention? | - Tell me about your experience with taking part in the D1 Now study? What was it like? | - How has your self-management been over the past 6/12 months? - How did the D1 Now study impact on your self-management? |
| **Intervention supports**  Florence | - What was it like using Florence? | - How well did you think the BG reminders worked? Why so? - How well did you think the alcohol safety reminders worked? Why so? - How well did you think the sick day rules reminders worked? Why so? - What did you think about the motivational messages? - What did you think about the amount of messages you were getting daily? |
| **Intervention supports**  **Agenda setting tool** | As you know, part of D1 Now was using the agenda setting tool in your clinic appointments.   - Can you tell me about your experience of using it? - What do you think about having a question about distress? | - What parts did you think were useful/not useful? - How could we improve the tool? - Tell me about your experience of doing the mapping exercise with your doctor |
| **Intervention supports**  **Support worker** | - Tell me about your experience of working with the Support Worker | - What worked well? Why? - What didn’t work so well? Why? - Did the Support Worker ring you in between appointments? What did you think about that? - As you may remember, the Support Worker was able to check how you were getting on with Florence. Can you tell me a bit about that? |
| **Intervention supports**  D1 Now | As you know the intervention consists of three pieces – Florence, the agenda setting tool and the support worker – together.   - How do you think they work together as a package? - What do you think about using them as standalones? | - What piece did you think worked well? Why? - What piece didn’t work so well? Why not? |
| **Feasibility** | You may remember as part of the study we asked you to complete a booklet of questionnaires at the beginning and end?   - Were there any questions that were particularly easy or difficult? | - Is there anything we could change in the booklet of questionnaires that you think might improve it? |
| **Covid Impact** | - Did Covid restrictions have any impact on your participation in the D1 Now study? | - Personal impact of Covid on diabetes management - Interaction with clinic and appointments |
| **Finish** | - Is there anything else you would like to share with me regarding your experiences of participating in the study? | - Is there anything I haven’t asked you that you wanted to talk about? |

**Healthcare Staff**

The D1 Now study in which you are taking part is a ‘pilot’ study. This means we are trying to learn as much as we can about which parts of the intervention work well and which don’t work so well before rolling it out on a larger scale. It is very important that we hear what doctors (healthcare staff think about the intervention and about their experiences of using D1 Now with their patients. So I’m very pleased that you’ve agreed to do this interview today. Just to remind you, we can stop the interview at any time if you want.

| **Topic** | **Question** | **Prompt** |
| --- | --- | --- |
| **Experience**  What are consultants’ experiences of using the D1 Now intervention? | Tell me about your experience with taking part in the D1 Now study? What was it like? | How did it impact on the YAs? |
| **Intervention supports**  Florence | What did you think about Florence? | How well did you think the BG **reminders** worked? Why so?  How well did you think the alcohol safety reminders worked? Why so?  How well did you think the sick day rules reminders worked? Why so?  What did you think about the motivational messages?  Tell me about your experience of using the clinician interface |
| **Intervention supports**  Agenda setting tool | How did you find the using the **agenda setting tool** in your appointments with the YAs?  What do you think about having a question about distress? | What parts did you think were useful/not useful?  How could we improve the tool?  Tell me about your experience of doing the mapping exercise with the YAs |
| **Intervention supports**  Support worker | What was it like having a support worker in the clinic? | **External**  What was your experience of having a new support person in the clinic?  How did you feel Support Worker fit into the existing clinic setup? What worked well?  What didn’t work so well? How could this process be improved ?  **Internal**  Tell me about how you decided on the appropriate person  How did you feel about the SW reducing time on usual nursing duties?  **Both**  How could we improve this role? |
| **Intervention supports**  D1 Now | As you know the intervention consists of three pieces together, do you think they work well as a package or would be better as standalones? | What piece did you think worked well? Why?  What piece didn’t work so well? Why not? |
| **Feasibility** | How feasible do you think it would be to implement this intervention in the clinic going forward?  Could you see this working long term in your clinic? | Is it acceptable to the clinic staff?  What do we need to change?  What can we do to make it more sustainable? |
| Finish | Do you have any other thoughts on your experience of taking part in this study? | Is there anything I haven’t asked you that you wanted to talk about? |
